# Lowering serum homocysteine in H-type hypertensive patients with atrial fibrillation after radiofrequency catheter ablation to prevent atrial fibrillation recurrence

**DOI:** 10.3389/fnut.2022.995838

**Published:** 2022-09-13

**Authors:** Youzheng Dong, Ting Huang, Zhenyu Zhai, Quanbin Dong, Zhen Xia, Zirong Xia, Jianhua Yu, Xinghua Jiang, Kui Hong, Yanqing Wu, Xiaoshu Cheng, Juxiang Li

**Affiliations:** ^1^Department of Cardiovascular Medicine, The Second Affiliated Hospital of Nanchang University, Nanchang, China; ^2^Jiangxi Key Laboratory of Molecular Medicine, Nanchang, China; ^3^Jiangxi Provincial Cardiovascular Disease Clinical Medical Research Center, Nanchang, China

**Keywords:** homocysteine, atrial fibrillation, radiofrequency catheter ablation, enalapril-folic acid tablet, recurrence

## Abstract

**Background:**

Prior investigation revealed that elevated serum total homocysteine (tHcy) are strongly correlated with atrial fibrillation (AF) recurrence. Herein, the goal of this study was to elucidate whether folic acid (FA) treatment reduced AF recurrence following radiofrequency catheter ablation (RFCA).

**Methods:**

To conduct this retrospective research, we included consecutive H-type hypertensive AF patients, who were treated with first RFCA, between January 2010 and January 2022. We assessed the AF recurrence risk between patients who were taking 10 mg enalapril and 0.8 mg FA in a single-pill combination (enalapril–FA) daily and those who were taking a pill of 10 mg enalapril only. Outcomes were compared using the propensity-score matched analysis. Cox regression model was employed for the evaluation of AF recurrence events.

**Results:**

Out of 2,714 patients, 645 patients receiving enalapril and 282 patients receiving enalapril-FA were included for analysis. Following propensity score matching, 239 patients remained in each group. These patients were followed-up for a median of 379 (137–596) days, and revealed that the enalapril-FA patients had drastically reduced AF recurrence, compared to the enalapril patients [adjusted hazard ratio (HR), 0.68; 95% confidence interval (CI), 0.48–0.97; *P* = 0.029]. Apart from this, no interactions were detected in the subgroup analysis.

**Conclusion:**

In H-type hypertensive AF patients who were treated with first RFCA, FA supplementation was correlated with a reduced AF recurrence risk.

## Introduction

The Global Burden of Disease Project reported that approximately 46.3 million people suffered from atrial fibrillation (AF) in 2016 (1). AF and associated disorders greatly impact patient quality of life, and generate a heavy financial burden on households and society. Although radiofrequency catheter ablation (RFCA) is a commonly used procedure of AF management, patients still has a high long-term recurrence rate and require re-ablation (2–5).

Homocysteine is a sulfur-based amino acid, and its circulating levels reflect the transsulphuration and transmethylation statuses of the human body (6). Multiple factors, including genetics, nutrition, lifestyle, diseases and certain medications contribute to the serum total homocysteine (tHcy) augmentation (7–9). Currently, based on the Chinese medical standards, serum tHcy levels over 10 μmol/L is defined as hyperhomocysteinemia. A hyperhomocysteinemic condition accompanied with hypertension is abbreviated as H-type hypertension (10). Emerging evidences suggested that elevated tHcy levels greatly increase stroke, dementia, atherosclerosis, hypertension, ischemic heart disease (IHD) and fetal malformation risks (11–14). Moreover, several studies have reported that a rise in tHcy levels correlate with an increased risk of AF recurrence in AF patients following sinus rhythm restoration (15–17).

Folic acid (FA), a water-soluble vitamin B9, serves as a cofactor in the Hcy remethylation pathway, which decreases serum tHcy levels, thereby diminishing the toxicity of Hcy (18). Multiple clinical studies demonstrated that FA supplementation reduces the risks of cardiovascular and cerebrovascular diseases including stroke, cognitive impairment, Alzheimer disease, IHD, myocardial infarction, and even death (19–21). Thus far, there are no reports on the relationship between FA supplementation and AF prognosis. Hence, this research explored the potential of FA administration in reducing AF recurrence in H-type hypertensive AF patients following RFCA.

## Methods

### Study design and population

This retrospective investigation included consecutive 2714 H-type hypertensive AF patients, who were treated with first RFCA at the Second Affiliated Hospital of Nanchang University between January 2010 and January 2022. Exclusion criteria were: (1) patient who did not take a daily dose of enalapril-FA (10 mg/0.8 mg) or enalapril (10 mg); (2) previous history of ablation and (3) cardiac pacemaker or defibrillator. Our work strictly followed the Declaration of Helsinki (2013), and obtained ethical approval from the Second Affiliated Hospital of Nanchang University.

### Radiofrequency ablation strategy

All patients received oral anticoagulation medication for a minimum of 4 weeks before RFCA. Antiarrhythmic drugs (AADs) were stopped for ≥5 half-lives prior to ablation. A decapolar catheter was positioned through the left femoral vein and into the coronary sinus. Next, a circumferential mapping catheter (Lasso, Biosense Webster, Diamond Bar, CA) was inserted into the pulmonary veins, and a 3.5 mm ablation electrode (Navistar Thermocool, Biosense Webster) was placed *via* the right femoral vein and into the left atrium (LA). Heparin was provided intraoperation for activated clotting time maintenance between 300 and 350 s. All patients initially received circumferential antral pulmonary vein isolation (PVI) 1–2 cm from the ipsilateral pulmonary vein (PV) ostia. PVI was conducted using the following parameters: 30–40 W power, 43°C highest temperature, and 30 ml/min irrigation rate. In paroxysmal AF patients, the endpoint of ablation was the elimination of ectopic triggers and the inability to re-induce AF. In presence of non-PV trigger, further isolation was carried out to eliminate the non-PV trigger. For example, in patients with enhanced signals or spontaneous ectopic activity from the superior vena cava (SVC), the SVC was isolated. Following trigger elimination, if patients with induced AF lasting >5 min, additional isolation was performed, namely, LA linear (LA roofline and mitral isthmus line) and complex fractionated atrial electrogram (CFAE) ablation at the physician's discretion. In patients with non-paroxysmal AF, the endpoint was to terminate AF and restore sinus rhythm. In non-paroxysmal AF after PVI, additional substrate modification was conducted at the physician's discretion. Substrate ablation consisted of LA linear and CFAE ablation. If the patient was still in the AF rhythm following the aforementioned ablation, then, external electrical cardioversion was instantly provided. In addition, in case of previously recorded classical AFL or recording during RFCA, ablation was performed according to the cavotricuspid isthmus (CTI). Oral anticoagulation was sustained following the ablation procedure and AADs for the following 3 months.

### Enalapril-FA or enalapril therapy

Hypertensive patients with preoperative serum tHcy level ≥10 μmol/L were administered with enalapril-FA (10 mg enalapril and 0.8 mg FA) immediately after RFCA, given that the patient consented to this treatment, and there were no contraindications. The decision of enalapril usage was made by the treating physicians.

### Patient follow-ups and study definitions

The patients were followed up at the 1-, 3-, 6-, 9-, and 12-month post operation using the 12-lead regular electrocardiogram (ECG) and 24-h Holter-ECG *via* outpatient review, as well as at every 3–6 months thereafter. The patients were advised to seek immediate medical attention if any AF recurrence symptom surfaced, such as, palpitations, dyspnea, fatigue, chest tightness/pain, poor effort tolerance, dizziness, syncope. Following 3 months, AADs were discontinued, while oral anticoagulation was maintained based on CHA2DS2-VASc. H-type hypertension was described as hypertension with a plasma tHcy ≥ 10 μmol/L. The baseline and final tHcy concentrations were collected. The primary endpoint was AF recurrence, which was defined as documented AF, atrial flutter, or atrial tachycardia >30 s following a blanking period of 3 months. If patients stopped taking the enalapril-FA or enalapril during the period of follow-up, due to poor tolerance or other reasons, then, their data were censored.

### Statistical analysis

The numeric variables were assessed *via* Student's t- or the rank-sum test, and the data were presented as means (standard deviation) or median (inter-quartile range). The comparisons of the categorical variables were assessed using χ2 or the Fisher's exact test, and the data were presented as percentages (%). To reduce potential bias between groups, we conducted propensity-score matching (PSM) to balance baseline characteristics. For the PSM, the 1:1 nearest neighbor matching was used without replacement, using a caliper width of 0.2. Standardized differences were used to assess the balance of matched groups, and absolute values >0.2 considered unacceptably imbalanced (22). For the propensity score–matched cohorts, we compared AF recurrence between enalapril-FA and enalapril using Kaplan–Meier and multivariate Cox regression models. Adjusted hazard ratio (HR) and 95% confidence interval (CI) were also calculated. The crude model was not adjusted; whereas, Model I was adjusted for age, sex, body mass index (BMI), smoking status, alcohol drinking status, AF duration, left atrial diameter (LAD), and AF type; and Model II was adjusted for covariates in Model I + systolic blood pressure (SBP), diastolic blood pressure (DBP), left ventricle ejection fraction (LVEF), estimated glomerular filtration rate (eGFR), serum creatinine (Scr), uric acid (UA), HbA1c, total cholesterol (TC), triglyceride (TG), high-density lipoprotein cholesterol (HDL-c), low-density lipoprotein cholesterol (LDL-c), brain natriuretic peptide (BNP), additional ablation, drugs, and history of diseases (coronary heart disease (CAD), diabetes, HF, hyperlipidaemia, and renal insufficiency). Proportional hazards assumptions were checked using Schoenfeld residuals. Moreover, subgroup analyses were performed using tests of interactions, and the adjusted HR and 95% CI within each subgroup were calculated and displayed in the forest plot. The pre-specified subgroups were based on patient age, sex, AF type, AF duration, CAD, diabetes, New York Heart Association (NYHA) class and additional ablation; and the adjustment variables were: BMI, smoking status, alcohol drinking status, SBP, DBP, eGFR, Scr, UA, BNP, HbA1c, TC, TG, HDL-c, LDL-c, LAD, LVEF, drugs, and history of diseases (HF, hyperlipidaemia, and renal insufficiency). Two-tailed *p* < 0.05 was set as the significance threshold. RStudio version 1.1.414 (Boston, MA, USA) and Empower (http://www.empowerstats.com; X&Y Solutions, Inc., Boston, MA) were employed for all data analyses.

## Results

### Characteristics of the study population

**Figure 1:**
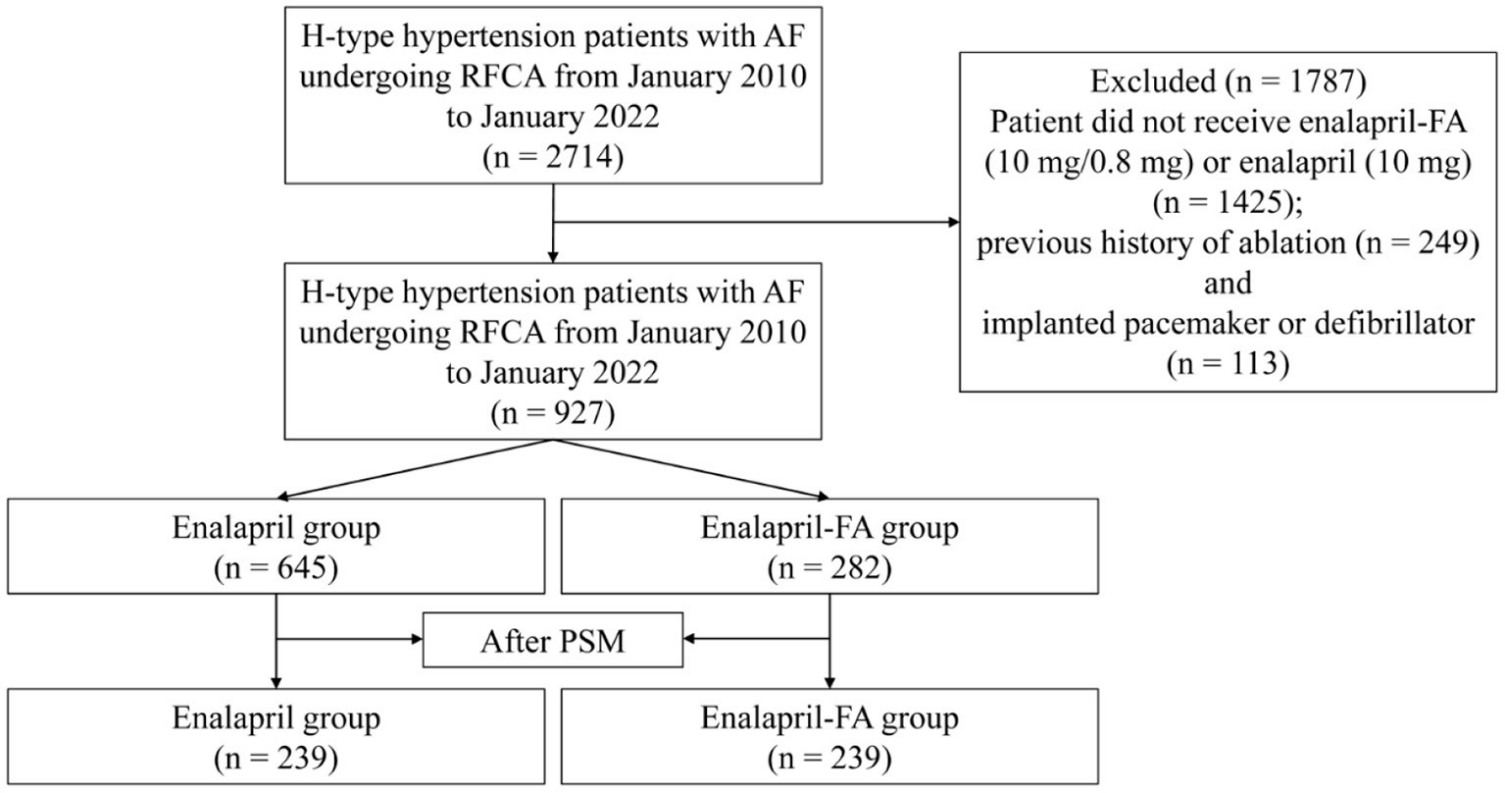
Study cohort flow diagram. AF, atrial fibrillation; RFCA, radiofrequency catheter ablation; FA, folic acid; PSM, propensity score matching.

Between 2010 1 and 2022 1, we screened 2,714 patients, and included 927 patients in analysis (Figure 1). Out of the 927 patients, 645 (69.58%) patients belonged to the enalapril group, and 282 (30.42%) belonged to the enalapril-FA group. Prior to PSM, we observed differences in certain baseline variables between the two groups, namely, in the age, sex, BMI, SBP, DBP, HDL-c, tHcy, HbA1c, eGFR, Scr, UA, BNP, LAD, LVEF, NYHA class, CHA2DS2-VASc score, drugs, history of hyperlipidaemia, CAD, stroke and renal insufficiency (Table 1). After PSM, there was no significant difference in baseline variables between the two groups. Supplementary Figure 1 illustrates the histogram of propensity score (PS) distribution, which is an evaluation of baseline balance, hence, it can be used to compare the score distribution similarities before and after matching, thereby providing an estimation of the common support domain area. Of the 239 patients in enalapril-FA group, the average age was 66.7 years, with 36.4% female population, average BMI of 25.1, and 55.2% of paroxysmal AF patients. In the enalapril group, the average age was 66.0 years, with 39.3% female population, average BMI of 25.0, and 57.3% of paroxysmal AF patients.

**Table 1:** Baseline characteristics of the study population.

**Characteristic**	**Before matching**			**After matching**		
	**Enalapril**	**Enalapril-FA**	***P-*value**	**Enalapril**	**Enalapril-FA**	***P-*value**
*N*	645	282		239	239	
Age (years)	63.05 (10.28)	67.32 (9.36)	<0.001	65.97 (9.51)	66.72 (9.32)	0.388
Female (%)	293 (45.43%)	97 (34.40%)	0.002	94 (39.33%)	87 (36.40%)	0.509
BMI (kg/m2)	24.46 (3.84)	25.14 (3.49)	0.011	24.98 (4.44)	25.11 (3.34)	0.708
AF type (%)			0.140			0.645
Paroxysmal	388 (60.16%)	155 (54.96%)		137 (57.32%)	132 (55.23%)	
Non-paroxysmal	257 (39.84%)	127 (45.04%)		102 (42.68%)	107 (44.77%)	
Duration of AF (months)	15.00 (6.00–36.00)	17.50 (6.00–40.75)	0.862	15.00 (4.50–36.00)	18.00 (6.00–37.00)	0.988
Current smoking (%)	143 (22.17%)	63 (22.34%)	0.954	51 (21.34%)	52 (21.76%)	0.911
Current alcohol (%)	103 (15.97%)	48 (17.02%)	0.690	39 (16.32%)	35 (14.64%)	0.613
Hyperlipidaemia (%)	127 (19.69%)	78 (27.66%)	0.007	64 (26.78%)	64 (26.78%)	1.000
Coronary artery disease (%)	107 (16.59%)	71 (25.18%)	0.002	54 (22.59%)	53 (22.18%)	0.913
Diabetes (%)	109 (16.90%)	57 (20.21%)	0.226	42 (17.57%)	47 (19.67%)	0.557
Heart failure (%)	131 (20.31%)	62 (21.99%)	0.933	51 (21.34%)	55 (23.01%)	0.660
Stroke (%)	84 (13.02%)	66 (23.40%)	<0.001	46 (19.25%)	46 (19.25%)	1.000
Renal insufficiency (%)	28 (4.34%)	56 (19.86%)	<0.001	24 (10.04%)	36 (15.06%)	0.098
SBP (mm Hg)	129.58 (19.30)	133.30 (19.15)	0.007	131.19 (19.78)	133.12 (19.11)	0.278
DBP (mm Hg)	76.27 (12.22)	79.45 (12.47)	<0.001	79.18 (12.91)	78.52 (12.33)	0.572
HR (beat per minute)	81.10 (27.63)	82.64 (26.81)	0.430	82.93 (28.58)	82.58 (27.44)	0.891
TC (mmol/L)	4.37 (1.09)	4.32 (0.98)	0.465	4.36 (1.06)	4.35 (0.92)	0.892
TG (mmol/L)	1.48 (0.94)	1.57 (1.03)	0.184	1.58 (1.05)	1.58 (1.04)	0.936
HDL-c (mmol/L)	1.18 (0.33)	1.13 (0.31)	0.022	1.17 (0.33)	1.13 (0.30)	0.118
LDL-c (mmol/L)	2.53 (0.84)	2.46 (0.73)	0.229	2.47 (0.80)	2.49 (0.71)	0.753
tHcy (μmol/L)	14.20 (2.46)	15.10 (8.83)	0.019	14.40 (2.52)	14.59 (6.75)	0.678
HbA1c	5.64 (0.72)	5.74 (0.65)	0.043	5.66 (0.76)	5.75 (0.62)	0.178
eGFR (mL/min per 1.73m2)	88.13 (19.19)	75.98 (22.81)	<0.001	80.92 (18.33)	78.39 (21.16)	0.163
Scr (umol/L)	76.65 (17.74)	94.48 (56.08)	<0.001	83.57 (20.30)	87.42 (23.85)	0.058
UA (umol/L)	355.36 (101.62)	398.28 (119.15)	<0.001	371.26 (113.98)	387.07 (114.05)	0.130
BNP (pg/ml)	135.06 (63.76–304.83)	164.36 (69.61–350.24)	0.002	165.00 (76.63–361.93)	145.34 (67.87–321.11)	0.744
LAD (mm)	40.27 (6.53)	41.54 (5.59)	0.005	40.98 (5.76)	41.43 (5.60)	0.389
LVEF (%)	60.55 (9.02)	58.25 (9.74)	<0.001	59.72 (9.90)	58.54 (9.51)	0.186
PV isolation (%)	645 (100%)	282 (100%)	0.509	239 (100%)	239 (100%)	1.000
SVC isolation (%)	123 (19.07%)	49 (17.38%)	0.542	38 (15.90%)	40 (16.74%)	0.804
LA CFAE ablation (%)	103 (15.97%)	37 (13.12%)	0.265	35 (14.64%)	32 (13.39%)	0.693
LA linear ablation (%)	279 (43.26%)	119 (42.20%)	0.765	108 (45.19%)	104 (43.51%)	0.713
CTI ablation (%)	202 (31.32%)	80 (28.37%)	0.369	77 (32.22%)	68 (28.45%)	0.371
NYHA functional class (%)			0.019			0.863
I	403 (62.48%)	145 (51.42%)		138 (57.74%)	129 (53.97%)	
II	187 (28.99%)	105 (37.23%)		80 (33.47%)	86 (35.98%)	
III	46 (7.13%)	27 (9.57%)		17 (7.11%)	19 (7.95%)	
IV	9 (1.40%)	5 (1.77%)		4 (1.67%)	5 (2.09%)	
CHA2DS2-VASc score	2.85 (1.42)	3.26 (1.58)	<0.001	3.13 (1.61)	3.15 (1.59)	0.864
Drugs (%)						
Beta-blockers	251 (38.91%)	128 (45.39%)	0.065	102 (42.68%)	106 (44.35%)	0.712
CCB	329 (51.01%)	169 (59.93%)	0.012	153 (64.02%)	149 (62.34%)	0.704
Statins	243 (37.67%)	144 (51.06%)	<0.001	118 (49.37%)	114 (47.70%)	0.714
MRA	69 (10.70%)	40 (14.18%)	0.129	24 (10.04%)	31 (12.97%)	0.316
Diuretics	102 (15.81%)	53 (18.79%)	0.263	36 (15.06%)	46 (19.25%)	0.225
Digoxin	57 (8.84%)	36 (12.77%)	0.067	23 (9.62%)	29 (12.13%)	0.378
Anticoagulation			0.002			0.111
Warfarin	31 (4.81%)	6 (2.13%)		13 (5.44%)	5 (2.09%)	
Dabigatran	413 (64.03%)	166 (58.87%)		131 (54.81%)	146 (61.09%)	
Rivaroxaban	177 (27.44%)	106 (37.59%)		88 (36.82%)	85 (35.56%)	
AADs			0.980			0.357
Amiodarone	463 (71.78%)	199 (70.57%)		177 (74.06%)	167 (69.87%)	
Propafenone	53 (8.22%)	26 (9.22%)		15 (6.28%)	24 (10.04%)	
Dronedarone	6 (0.93%)	2 (0.71%)		0 (0.00%)	2 (0.84%)	
Sotalol	6 (0.93%)	3 (1.06%)		3 (1.26%)	3 (1.26%)	
Follow-up (days)	302.00 (134.00–556.00)	413.00 (140.25–615.50)	0.200	348.00 (135.00–538.50)	412.00 (139.00–621.50)	0.492

Data are presented as mean (standard deviation), or median (IQR) and percentages.

FA, folic acid; BMI, body mass index; AF, atrial fibrillation; SBP, systolic blood pressure; DBP, diastolic blood pressure; HR, heart rate; HbA1c, hemoglobin A1c; TC, total cholesterol; TG, triglyceride; HDL-c, high density lipoprotein cholesterol; LDL-c, low density lipoprotein cholesterol; tHcy, total homocysteine; eGFR, estimated glomerular filtration rate; Scr, serum creatinine; UA, uric acid; BNP, brain natriuretic peptide; LAD, left atrial diameter; LVEF, left ventricle ejection fraction; PV, pulmonary vein; SVC, superior vena cava; LA, left atrium; CFAE, complex fractionated atrial electrogram; CTI, cavotricuspid isthmus; NYHA, New York Heart Association; CCB, calcium channel blockers; MRA, mineralocorticoid receptor antagonist; AADs, antiarrhythmic drugs.

### Effects of FA supplementation on tHcy levels

**Figure 2:**
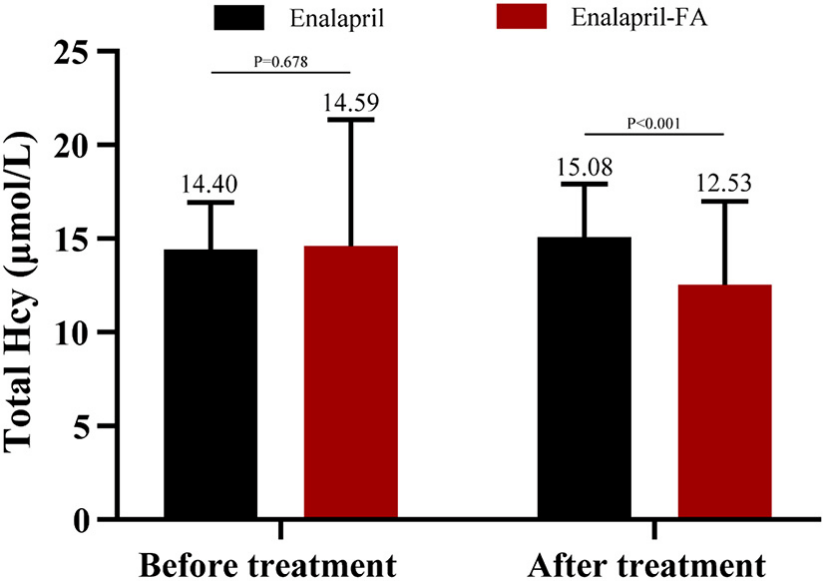
Mean plasma levels of tHcy before and after treatment in matched cohort. Plasma tHcy levels were obtained in 239 patients in the enalapril group and 239 patients in the enalapril-FA group before treatment; in 177 and 216, respectively, after treatment. tHcy, total homocysteine; FA, folic acid.

Figure 2 illustrates the serum tHcy levels of matched patient cohorts. At baseline, the tHcy levels showed no obvious differences between the two groups (14.40 ± 2.52 μmol/L vs. 14.59 ± 6.75 μmol/L; *P* = 0.678). After treatment, the levels of tHcy was significantly lower in the enalapril-FA group as compared to enalapril group (12.53 ± 4.45 μmol/L vs. 15.08 ± 2.83 μmol/L; *P* < 0.001).

### Primary endpoint

**Figure 3:**
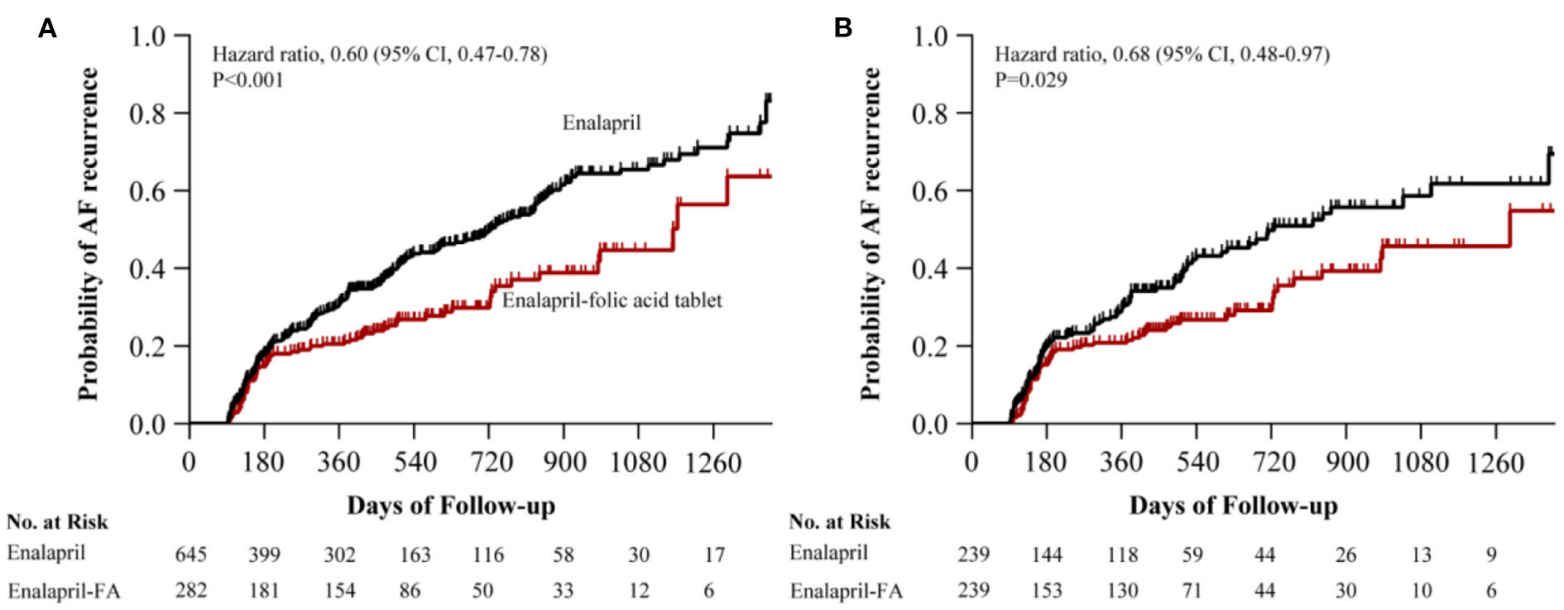
Kaplan–Meier curves of cumulative probability of AF recurrence before PSM **(A)** and after PSM **(B)**. The HR after PSM was adjusted for age, sex, BMI, duration of AF, LAD, AF type, smoking status, alcohol drinking status, SBP, DBP, eGFR, Scr, UA, HbA1c, TC, TG, HDL-c, LDL-c, BNP, LVEF, additional ablation, beta-blockers, CCB, statins, MRA, diuretics, digoxin, AADs and history of disease (CAD, diabetes, HF, hyperlipidaemia, renal insufficiency). AF, atrial fibrillation; PSM, propensity score matching; HR, hazard ratio; CI, confidence interval; FA, folic acid; BMI, body mass index; LAD, left atrial diameter; SBP, systolic blood pressure; DBP, diastolic blood pressure; eGFR, estimated glomerular filtration rate; Scr, serum creatinine; UA, uric acid; TC, total cholesterol; TG, triglyceride; HDL-c, high-density lipoprotein cholesterol; LDL-c, low-density lipoprotein cholesterol; BNP, brain natriuretic peptide; LVEF, left ventricle ejection fraction; CCB, calcium channel blocker; MRA, mineralcorticoid recept antagonist; AADs, antiarrhythmic drugs; CAD, coronary heart disease; HF, heart failure.

**Figure 4:**
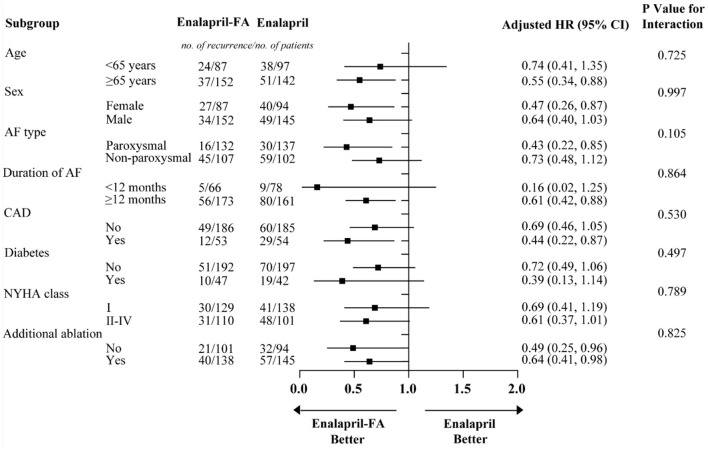
Subgroup analysis for AF recurrence rates in patients with enalapril-FA and enalapril. Models were adjusted for BMI, smoking status, alcohol drinking status, SBP, DBP, eGFR, Scr, UA, BNP, HbA1c, TC, TG, HDL-c, LDL-c, LAD, LVEF, beta-blockers, CCB, statins, MRA, diuretics, digoxin, AADs and history of diseases (HF, hyperlipidaemia and renal insufficiency). FA, folic acid; AF, atrial fibrillation; BMI, body mass index; SBP, systolic blood pressure; DBP, diastolic blood pressure; eGFR, estimated glomerular filtration rate; Scr, serum creatinine; UA, uric acid; BNP, brain natriuretic peptide; TC, total cholesterol; TG, triglyceride; HDL-c, high-density lipoprotein cholesterol; LDL-c, low-density lipoprotein cholesterol; LAD, left atrial diameter; LVEF, left ventricular ejection fraction; CCB, calcium channel blocker; MRA, mineralcorticoid recept antagonist; AADs, antiarrhythmic drugs; HF, heart failure; HR, hazard ratio; CI: confidence interval.

Prior to PSM, after a median follow-up of 344 (136–590) days, AF recurrence was present in 73 (25.9%) patients in the enalapril-FA group and 256 (39.7%) patients in the enalapril group. Enalapril-FA treatment significantly lowered AF recurrence risk, compared to enalapril (HR, 0.60; 95% CI: 0.47–0.78; *P* < 0.001; Figure 3A). Following PSM, AF recurrence occurred in 61 (25.5%) patients with enalapril-FA vs. 89 (37.2%) patients with enalapril by the end of a median 379 (137–596) days follow-up. Moreover, relative to the enalapril group, the crude HR and adjusted HR for AF recurrence in the enalapril-FA group were 0.65 (95% CI: 0.47–0.90; *P* = 0.009; crude model), 0.65 (95% CI: 0.47–0.91; *P* = 0.011; model I) and 0.68 (95% CI: 0.48–0.97; *P* = 0.029; model II, Figure 3B; Table 2), respectively. Figure 4 depicts the relationships between the enalapril-FA and AF recurrence in multiple clinical subgroups. None of the examined interactions reached significance.

**Table 2:** Risk of AF recurrence in the Propensity-Score–Matched Cohort.

			**Crude**		**Model I**		**Model II**	
	**No. of Patients with event**	**Event rate**	**HR (95% CI)**	***P***	**HR (95% CI)**	***P***	**HR (95% CI)**	***P***
Enalapril	89	37.24%	Ref.		Ref.		Ref.	
Enalapril-FA	61	25.52%	0.65 (0.47, 0.90)	0.009	0.65 (0.47, 0.91)	0.011	0.68 (0.48, 0.97)	0.029

Model I was adjusted for age, sex, BMI, smoking status, alcohol drinking status, duration of AF, LAD and AF type; Model II was adjusted for age, sex, BMI, duration of AF, LAD, AF type, smoking status, alcohol drinking status, SBP, DBP, Scr, UA, HbA1c, total cholesterol, triglyceride, HDL-c, LDL-c, BNP, LVEF, additional ablation, NYHA functional class, beta-blockers, CCB, statins, MRA, diuretics, digoxin, AADs and history of disease (hyperlipidaemia, diabetes, coronary heart disease, heart failure, stroke and renal insufficiency).

HR, hazard ratio; 95% CI, 95% confidence interval; FA, folic acid; BMI, body mass index; AF, atrial fibrillation; LAD, left atrial diameter; SBP, systolic blood pressure; DBP, diastolic blood pressure; Scr, serum creatinine; UA, uric acid; HbA1c, hemoglobin A1c; HDL-c, high-density lipoprotein cholesterol; LDL-c, low-density lipoprotein cholesterol; BNP, brain natriuretic peptide; LVEF, left ventricle ejection fraction; NYHA, New York Heart Association; CCB, calcium channel blockers; MRA, mineralocorticoid receptor antagonist; AADs, antiarrhythmic drugs.

## Discussion

This investigation is the first to assess the effectiveness of FA on AF recurrence in H-type hypertensive patients with AF following their first ablation. We demonstrated that the FA supplementation was linked to a reduced AF recurrence risk following a 3-month blanking period.

Worldwide, RFCA of AF is a very popular therapeutic strategy in the department of cardiology. However, the risk of AF recurrence remains high in patients with persistent AF following the initial ablation, and patients often need secondary ablation (23). Early identification and control of risk factors are crucial to preventing AF recurrence following ablation in the management of AF patients. In a study by Yao et al. (15), tHcy concentrations were markedly enhanced in patients with early recurrence of atrial tachyarrhythmia following RFCA, relative to those without early recurrence (15.1 ± 4.1 vs. 12.4 ± 3.7 mmol/L, *P* < 0.001). Moreover, multivariate analysis revealed that the tHcy levels were strongly associated with early recurrence (OR 1.188, 95% CI 1.097–1.286, *P* < 0.001). Naji et al. (16) studied persistent AF after successful electrical conversion, demonstrating that the baseline tHcy levels > 14.4 μmol/L accurately estimate AF recurrence during a 1.5-year follow-up period. Nasso et al. (17) reported in patients with non-valvular AF that, following a minimally invasive epicardial ablation, increased tHcy could independently estimates AF recurrence, and the threshold of 16 μmol/L for Hcy generated a better diagnostic performance with area under the curve of 0.807. However, as a newly identified risk factor for AF recurrence, Hcy has not yet been adequately appreciated and deeply explored. Therefore, it is particularly important to understand the metabolic processes of Hcy and explore the Hcy-mediated mechanism of action in AF recurrence.

Hcy is metabolized through three major pathways (24): (1) FA absorbed from the gut is first reduced to tetrahydrofolate (THF), which then combines with methyls from the methyl pool (include dimethylglycine (DMG), sarcosine, serine and so on) to form 5,10 methylenetetrahydrofolate (5,10-MTHF). Next, 5,10-MTHF reductase (MTHFR) irreversibly catalyzes the conversion of 5,10-MTHF into 5-MTHF. This chemical reaction offers a methyl group to Hcy to form methionine, which is catalyzed by methionine synthetase (MS) and vitamin B12, which serves as a coenzyme; (2) Glycine betaine, known as Trimethylglycine (TMG), provides a methyl group to Hcy to form methionine under the catalysis of betaine homocysteine methyl (BHMT). In this process, DMG is formed, which is then employed in the methyl pool; (3) Hcy is catalyzed by cystathionine-β-synthase (CBS) to generate cystathionine and then cysteine. This process requires vitamin B6 as a cofactor. Cysteine can be further metabolized to form other important sulfur-containing compounds.

At present, multiple factors were found to promote the elevation of Hcy levels. Among the genetic factors are the deactivation of crucial enzymes required for Hcy metabolism, such as, MTHFR, CBS, MS and BHMT (25). Unhealthy life styles (smoking, consuming alcohol products, and lack of exercise) as well as dietary pattern could result in excessive consumption or low absorption of FA, vitamin B6, and vitamin B12 (26). Renal dysfunction, malignant tumors as well as methotrexate, antiepileptic drugs, and diuretics usage can increase the Hcy levels (27). In addition, age and the male gender are also significantly associated with increased Hcy (28).

Under the influence of risk factors, cardiac structural and electrical remodeling will be progressed gradually when the levels of Hcy beyond the whole-body metabolic capacity. Structural remodeling is a complex process, which includes cardiomyocyte hypertrophy and proliferation, as well as changes in components of extracellular matrix (ECM). It is well known that collagen I and III are major components of ECM in the atrial interstitium (29). Elevated Hcy levels might increase the collagen/elastin ratio by activating the extracellular signal regulated kinase (ERK) and matrix metalloproteinase-9 (MMP-9) signal axis, which results in ECM remodeling (30). Similarly, Masayuki Shimano et al. also reported that the Hcy levels were positively associated with the carboxy-terminal telopeptide of collagen type I (CITP) and LAD (31). Electrical remodeling, was defined as the impairment of electrical activation caused by molecular defects in cardiomyocytes, and it is known to be critical in promoting AF pathogenesis (32). An increase in Hcy levels could induce the electrical remodeling of ion channels in the atrial myocardium. This includes both the aberrant activation and inactivation of Na^+^ currents, enhanced L-type Ca^2+^ currents and inward rectifier K^+^ currents, as well as decreased transient outward and ultrarapid delayed rectifier K^+^ currents (33–35). Moreover, high Hcy levels also result in significant alterations in the atrial action potentials, which include a more hyperpolarized resting potential, enhanced plateau potential, and abbreviated action potential duration (APD), and eventually facilitate AF induction (36).

Being essential upstream mediators of atrial remodeling, oxidative stress (OS) and inflammatory responses play an important role in the occurrence and maintenance of AF (37, 38). The sulfhydryl groups contained in Hcy participate in the important oxidoreduction reactions in the body, which cause OS (39). Moreover, *via* the electron transport chain component leakage due to mitochondrial damage and activation of protein kinase-like endoplasmic reticulum kinase, Hcy can induce endothelial cell apoptosis and the phosphorylation of nuclear factor- kB (NF-kB), thus, generating a large amount of reactive nitrogen species (RNS) and reactive oxygen free radicals, which causes endothelial injury and dysfunction (39, 40). In the meantime, Hcy can also promote lipid peroxidation and intracellular calcium overload via nicotinamide adenine dinucleotide phosphate (NADPH) oxidase activation, which, in turn, induces atrial electrical and structural remodeling (39). Alongside these, increased Hcy is accompanied with upregulation of several pro-inflammatory cytokines, namely, Interleukin-1β, Interleukin-6, tumor necrosis factor α, monocyte chemotactic proteins 1, and intracellular adhesion molecule-1, which initiate the inflammation cascade and affect the occurrence and development of AF (39, 41).

Although the mechanism whereby FA improves AF recurrence are not fully clear, we believe that the FA acts as a methyl vector to correct the abnormality of methylation and transsulfuration, thereby decreasing the cardiomyocyte toxicity caused by Hcy, thus, reversing the cardiac structural and electrical remodeling. Further fundamental researches are warranted to elucidate the associated mechanisms.

This study had several limitations. First, the FA content of each enalapril-FA was 0.8 mg, which was a fixed dose, and we did not explore other FA dosages. Second, the AF recurrence rate may be under-reported as some patients may have experienced asymptomatic AF recurrence, and our follow-up plan did not include long-term ECG monitoring. Lastly, this investigation was a retrospective analysis, and therefore, further randomized controlled studies are needed.

In conclusion, we demonstrated that, in H-type hypertensive AF patients who were treated with first RFCA, FA supplementation was correlated to a reduced AF recurrence risk after a blanking period of 3 months.

## Data availability statement

The raw data supporting the conclusions of this article will be made available by the authors, without undue reservation.

## Ethics statement

The studies involving human participants were reviewed and approved by The Second Affiliated Hospital of Nanchang University Medical Research Ethics Committee. Written informed consent for participation was not required for this study in accordance with the national legislation and the institutional requirements.

## Author contributions

YD participated in the data analysis, data interpretation, and wrote the manuscript. TH and ZZ collected the data. QD, ZhX, ZiX, JY, XJ, KH, YW, and XC conceived the study and participated in its design and coordination. JL participated in the study design and provided critical revision. All the authors read and approved the final version of the manuscript.

## Funding

This study was supported by the Key Research and Development Program of Jiangxi Province (No. 20202BBGL73069), Second Affiliated Hospital of Nanchang University Funded Clinical Research Projects (No. 2021efyC01) and General Science and Technology Program of Jiangxi Provincial Health Commission (No. 202210627).

## Conflict of interest

The authors declare that the research was conducted in the absence of any commercial or financial relationships that could be construed as a potential conflict of interest.

## Publisher's note

All claims expressed in this article are solely those of the authors and do not necessarily represent those of their affiliated organizations, or those of the publisher, the editors and the reviewers. Any product that may be evaluated in this article, or claim that may be made by its manufacturer, is not guaranteed or endorsed by the publisher.

## Supplementary material

Supplementary Figure 1 The histogram of propensity score (PS) distribution in raw cohort **(A)** and matched cohort **(B)**. **(B)** Shows that the PS distribution of the enalapril-FA group was similar to that of the enalapril group.
