# Supplementary figures and images for: Lowering serum homocysteine in H-type hypertensive patients with atrial fibrillation after radiofrequency catheter ablation to prevent atrial fibrillation recurrence
Source: Front Nutr. 2022 Sep 13;9:995838. doi: 10.3389/fnut.2022.995838 (PMC9514121; doi:10.3389/fnut.2022.995838)

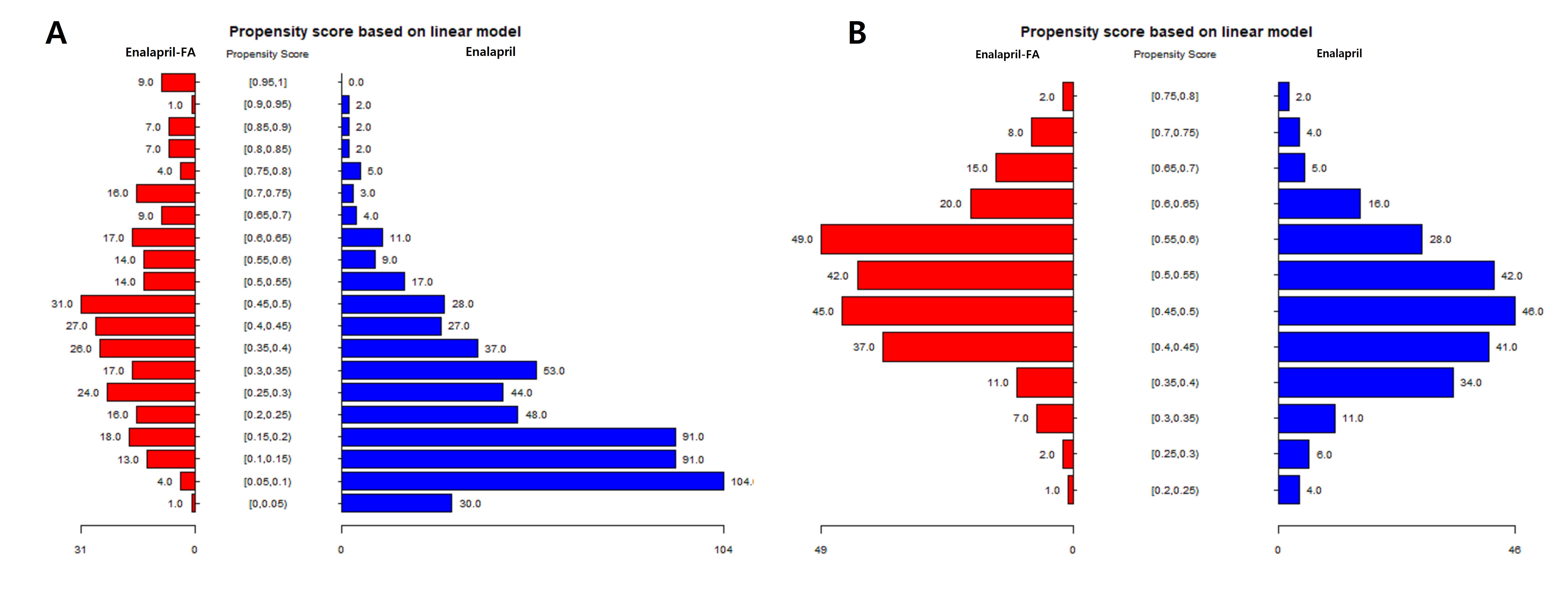

Supplement: Supplementary Figure 1 — The histogram of propensity score (PS) distribution in raw cohort (A) and matched cohort (B). (B) Shows that the PS distribution of the enalapril-FA group was similar to that of the enalapril group. [file Image_1.TIF]
